# Effect of Thin-Walled Radial Sheath for Large-Bore Access On Reducing Periprocedural Radial Artery Occlusion Following Complex PCI: The REDUCE-RAO Randomized Trial

**DOI:** 10.31083/j.rcm2310329

**Published:** 2022-09-28

**Authors:** Hao Wang, Hao-Yu Wang, Shao-Yu Wu, Dong Yin, Lei Feng, Wei-Hua Song, Hong-Jian Wang, Cheng-Gang Zhu, Ke-Fei Dou

**Affiliations:** ^1^Department of Cardiology, Cardiometabolic Medicine Center, Coronary Heart Disease Center, Fuwai Hospital, National Center for Cardiovascular Diseases, Chinese Academy of Medical Sciences and Peking Union Medical College, 100037 Beijing, China; ^2^State Key Laboratory of Cardiovascular Disease, 10037 Beijing, China

**Keywords:** 7-Fr Glidesheath Slender, complex PCI, large bore, radial artery occlusion, transradial access

## Abstract

**Background:**

Transradial artery (TRA) access for percutaneous coronary intervention (PCI) was associated with lower risks of major bleeding and vascular complications compared to transfemoral artery access. Use of large-bore (≥7-Fr) guiding catheters through TRA approach increased the likelihood of radial artery occlusion (RAO). This study aimed to investigate whether use of the thin-walled 7-Fr Glidesheath Slender, allowing PCI with large-caliber guiding catheters, is superior to standard 7-Fr Cordis sheath with respect to periprocedural RAO within 24 hours after transradial coronary intervention (TRI) in complex lesions.

**Methods:**

A prospective randomized, controlled, single-blinded (patient-blinded) trial was conducted, randomizing 504 patients with TRI for complex lesions to either 7-Fr Glidesheath Slender or conventional 7-Fr Cordis sheath. The primary outcome was defined as the incidence of periprocedural RAO with Doppler ultrasound during the first 24 hours after TRI.

**Results:**

The incidence of early RAO was 10.3% for 7-Fr Glidesheath Slender and 13.5% for conventional 7-Fr sheath (*p* = 0.271). The procedural success rate for Glidesheath Slender was 92.9% and for Cordis sheath was 93.7% (*p* = 0.722). There was no signficiant difference between treatment arms in terms of local hematoma and radial spasm, whereas use of the Glidesheath Slender was associated with significantly less pain during the procedure (numeric rating scale [NRS], 2.27 ± 0.75 vs. 2.45 ± 0.95, *p* = 0.017). The assessment of radial artery in ultrasound parameters after complex TRI was improved with Glidesheath Slender.

**Conclusions:**

Among patients with complex coronary lesions undergoing TRI, 7-Fr Glidesheath Slender was not superior to conventional 7-Fr in the prevention of periprocedural RAO within 24 hours following complex PCI, without reducing RAO occurrence.

**Clinical Trial Registration:**

NCT04748068.

## 1. Introduction

Transradial access (TRA) has been recommended as the default approach for diagnostic and interventional cardiovascular procedures because of reduced risk of vascular and bleeding complications as well as lower rates of mortality and increased patient comfort [1]. TRA has been shown to be endorsed as the preferable access strategy in patients undergoing coronary angiography and/or percutaneous coronary intervention (PCI), with growing recommendations in guidelines [2, 3, 4, 5, 6]. Altough the risk of complications with TRA are lower than with the transfemoral access (TFA) approach, and the most frequent complication of TRA approach is radial artery occlusion (RAO) [7]. Early detection of peri-procedural RAO (within 24 hours) after PCI and preserving radial artery patency is of fundamental clinical importance as RAO hampers the use of the radial artery for future catheterization procedures. At present, large-sized sheath and guiding catheter (≥7-Fr) can be applied in the treatment of complex PCI, such as PCI for left main disease, complex bifurcation, chronic total occlusion (CTO), or heavily calcified lesions [8, 9]. It should be noted that even though the mounting “radial-first” strategy gained increased attention, the transition from TFA to TRA approach may be challenging in this clinical scenario, as use of large-bore guiding catheters (≥7-Fr) through TRA was associated with higher risks of RAO [10, 11, 12]. The development of a thin-walled radial introducer sheath (7-Fr Glidesheath Slender), allowing PCI with large-caliber guiding catheters, supported the preliminary use of TRA also in complex PCI. However, it is unclear whether the routine use of 7-Fr Glidesheath Slender will result in reduction of early RAO compared with the commonly used 7-Fr Cordis sheath in patients who underwent for complex coronary anatomy throught TRA. Therefore, we performed a prospective randomized, single-blind superiority trial, comparing the rate of peri-procedural RAO (within 24 hours) after complex PCI between the 7-Fr Glidesheath Slender and the 7-Fr Cordis conventional sheath with TRA.

## 2. Materials and Methods

### 2.1 Study Design

The REDUCE-RAO randomized (effect of thin-walled radial sheath for large-bore access on REDUCing pEriprocedural Radial Artery Occlusion following complex PCI) trial was a prospective, single-blind, randomized controlled study that was initiated by the investigator (ClinicalTrials.gov identifier: NCT04748068), in which eligible subjects were randomly assigned in 1:1 fashion to receive either a “7-Fr Glidesheath Slender” or a “7-Fr Cordis sheath”. The present study was approved by the Fuwai Hospital Institutional Review Board. This study followed the Declaration of Helsinki. Before enrollment, all patients gave written informed consent.

### 2.2 Objectives

The purpose of this trial was to investigate whether 7-Fr Glidesheath Slender is superior to 7-Fr Cordis conventional sheath in patients undergoing complex transradial PCI with respect to periprocedual RAO within 24 hours. As secondary objectives, 7-Fr TRA access with Glidesheath Slender and Cordis sheath for transradial PCI were compared with regard to vascular access site complications, radial artery spasm, procedure success, and the degree of pain at the puncture site.

### 2.3 Inclusion and Exclusion

Adults (≥18 years) in whom PCI was planned for complex chronic total occlusion (CTO), heavily calcified lesions (required a rotablator system), complex bifurcation, or left main stem based on angiographic assessment were considered for randomization. Patients were assessed for inclusion if the operator thought a 7-Fr guiding catheter would be needed. The complex bifurcation defined as two-stent strategy or kissing balloon inflation in left main bifurcation lesions, and need for active side branch protection strategy. Further eligibility criteria were stable or unstable angina pectoris; or those ≥72 hours post-myocardial infarction before screening. Before PCI, a radial artery ultrasound were performed to document patency of the radial artery. Principal exclusion criteria were as follows: (1) patients with RAO before PCI; (2) patients in cardiac arrest, pulmonary edema, AND cardiogenic shock; (3) patients who had known bleeding disorder or hypercoagulable condition.

### 2.4 Description of the Sheaths 

As the first commercially available 7-Fr thin-walled sheath in China, the 7-Fr Glidesheath Slender (Terumo, Japan) has an inner diameter (ID) of 2.46 mm, compatible with other 7-Fr guiding catheters, with a reduced outer diameter (OD) of 2.79 mm (**Supplementary Fig. 1**). The 7-Fr Cordis sheath (Avanti+ Catheter Sheath Introducer, Cordis, USA, outer diameter: 3.02 mm) is a standard 7-Fr compatible sheath which is traditionally used in clinical practice in our center.

### 2.5 Procedures

Local subcutaneous anesthetic with 2% lidocaine was administered to the wrist, 1 to 2 cm proximal to the styloid process of the radial bone, after skin preparation. After placing the introducer 6-Fr sheath (Avanti+ Transradial Kit, Cordis, USA, outer diameter: 2.67 mm), heparin (5000 U) was given as an intra-arterial bolus through the radial sheath. Current standard procedures and recommendations were used to perform transradial coronary angiography.

If 7-Fr guiding catheter was decided to be used by the operator, patients received an intra-arterial injection of 200 μg nitroglycerin. Then initially inserted 6-Fr sheath was exchanged for the 7-Fr one (Glidesheath Slender or Cordis sheath according to patient randomization). An additional dosage of UFH was given in cases of PCI to bring the total dose to 125 IU/kg. After the process started, the dose of UFH was adjusted based on the activated clotting time monitoring (250–350 s). After the procedure, radial artery hemostasis was achieved with a locally placed compression device (Tourniquet, Helix) by a staff member not aware of the randomization group (**Supplementary Fig. 2**). Pulsation of the distal radial artery was required as much as possible. To maintain hemostasis without bleeding, special care was taken to achieve the lowest pressure possible and and followed by gradual decompression. After 6 hours post-PCI, the compression device was fully released and removed in both groups. The characteristics of the radial artery were evaluated using the duplex Doppler ultrasound (GE Vivid, the USA) (**Supplementary Fig. 3**) [13], performed in all patients before PCI and within 12–24 hours after TRI. Peri-procedural RAO was defined by the absence of anterograde flow on color Doppler ultrasound within 12–24 hours after TRI.

### 2.6 Endpoints and Definitions 

The primary end point was the incidence of peri-procedural RAO (within 24 hours) after complex TRI. Secondary end points were procedural success, vascular access-site complications and pain score (visual analog pain scale 0–10). Visual estimation of a final diameter stenosis of 50% and postprocedural TIMI flow grade 3 in all treated lesions were used to define procedural success. Vascular access-site complications included radial artery spasm, pseudoaneurysm, arteriovenous fistula and local hematoma. Radial artery spasm was described as pain in the forearm that was greater than 6 on a scale of 0 to 10 (numeric pain rating scale) during the procedure, in which patients were asked to state their maximal perceived pain during sheath insertion. An independent clinical event committee whose members were unaware of clinical, angiographic, and procedural data adjudicated all events.

### 2.7 Statistical Analysis

In a prospective multicenter study [14], the incidence of 1-month RAO in patients undergoing complex PCI using the TRA method with the 7-Fr Glidesheath slender was 4.8%. Based on previously studies, incident RAO ranged between 8.5% and 19% through the TRA approach using the standard 7-Fr sheath and varied with timing of assessment of radial artery patency [14, 15, 16]. In the present study, the proportion of patients with early RAO was assumed to be 12% in the standard 7-Fr Cordis sheath group and 5% in the 7-Fr Glidesheath Slender group. Randomizing 496 recruited patients with a 2-sided level of 0.05 and a maximum 0% rate of loss to follow-up would provide 80 percent power to establish superiority of the 7-Fr Glidesheath Slender over the 7-Fr Cordis sheath. The mean and standard deviation of continuous data are presented and compared using the Student’s *t*-test. Categorical variables were summarized as counts and percentages, and Chi-square or Fisher’s exact tests were used to compare them.To find the possible factors linked to the occurrence of early RAO after TRI, we adopted univariable and multivariable logistic regression analysis. The baseline variables with P0.10 in the univariable analysis, as well as any other baseline factors assessed to be of clinical relevance from previously published literature, were included in a multivariable logistic regression analysis using a backward stepwise method, including body mass index (BMI), diabetes, previous TRI, peripheral artery disease, heparin anticoagulation, procedure duration, successful PCI, ratio of the sheath outer diameter to the radial artery inner diameter (S/A), calcium channel blockers (CCB) use, statins use, compression time, preoperative radial artery diameter, volume of blood flow, and 7-Fr Glidesheath Slender. All statistical analyses were conducted in both the intention-to-treat (ITT) population and the as-treated set (ATS). SPSS version 23.0 (SPSS Inc., Chicago, IL, USA) was used for all statistical analyses, and a two-tailed *p *< 0.05 was considered statistically significant.

## 3. Results

### 3.1 Study Population

**Fig. 1.**
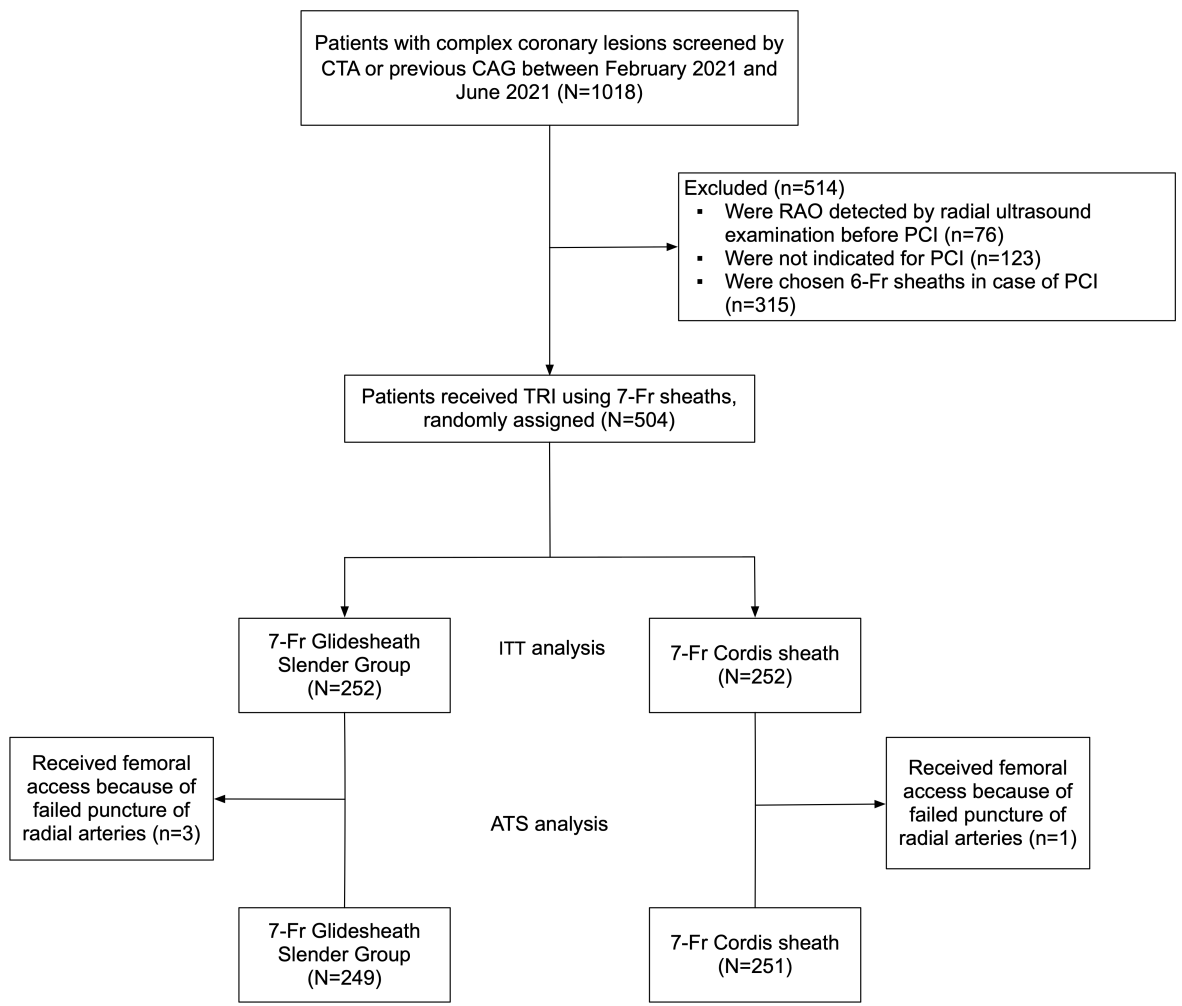
**Study Flowchart**. CTA, CT angiography; CAG, coronary angiography; RAO, radial artery occlusion; ITT, intention-to-treat; ATS, as-treated set.

From February 2021 to June 2021, previewed through CT angiography or previous coronary angiography, 1018 patients with complex coronary artery disease who would need a 7-Fr guiding catheter before PCI were assessed using radial artery ultrasonography. 76 patients were excluded due to RAO discovered by ultrasonography prior to PCI, 123 patients who did not require interventional therapy, and 315 patients were excluded due to the use of 6-Fr guidance. For the ITT set, a total of 504 patients were included and randomized to either 7-Fr TRA with 7-Fr Glidesheath Slender group (N = 252) or standard 7-Fr Cordis sheath group (N = 252). During PCI procedure, 3 patients in 7-Fr Glidesheath Slender group converted to femoral access for forearm artery deformity and 1 patient in 7-Fr Cordis sheath group converted to femoral access for sheath insertion failure. For the ATS, 249 patients underwent complex PCI with 7-Fr Glidesheath Slender, and 251 patients underwent complex PCI with 7-Fr Cordis sheath (Fig. 1).

### 3.2 Clinical and Procedural Characteristics

The baseline characteristics of the research population are shown in Table 1. There were no significant differences between the groups in terms of demographics (age, gender, and BMI), coronary artery disease risk factors (diabetes mellitus, dyslipidemia, and hypertension), clinical presentation, previous TRI history, or medication during hospitalization. The most commonly treated complex coronary lesions were CTO (44.4%), followed by bifurcation lesions (23.0%), severe tortuosity (15.3%), severe calcification (12.3%), and left main disease (5.0%) (Central Illustration). The ratio of sheath outer diameter and radial artery inner diameter (S/A) in 7-Fr Glidesheath Slender group was significantly lower than that in 7-Fr Cordis sheath group (1.12 ± 0.20 vs. 1.22 ± 0.22, *p *< 0.001). Additionally, the systolic pressure in sheath in Cordis sheath group was much higher than that of Glidesheath Slender group (148.2 ± 22.1 vs. 143.7 ± 24.1 mmHg, *p* = 0.032). There were no significant differences in lesion characteristics, heparin dose, anticoagulant medication, contrast medium volume, or compression time.

**Table 1.** **Clinical, Angiographic and Procedural characteristics**.

Variable		7-Fr Cordis conventional sheath (n = 252)	7-Fr Glidesheath Slender (n = 252)	*p* value
Age (years)		58.1 ± 10.4	57.4 ± 10.5	0.410
Male		209 (82.9)	216 (85.7)	0.391
BMI (kg/$m^{2}$)		26.0 ± 3.1	25.8 ± 2.9	0.468
Hypertension		163 (64.7)	151 (59.9)	0.270
Diabetes mellitus		78 (31.0)	92 (36.5)	0.187
Hyperlipidemia		247 (98)	250 (99.2)	0.258
Current smoker		135 (53.6)	151 (59.9)	0.150
Peripheral artery disease		14 (5.6)	18 (7.1)	0.465
Previous myocardial infarction		65 (25.8)	65 (25.8)	1.000
Previous PCI		87 (34.5)	105 (41.7)	0.099
Previous CABG		2 (0.8)	1 (0.4)	0.563
Previous stroke		22 (8.7)	18 (7.1)	0.510
Previous TRI		149 (59.1)	163 (64.7)	0.199
Systolic pressure		135.2 ± 17.5	134.3 ± 16.1	0.544
Diastolic pressure		79.0 ± 11.6	79.2 ± 11.2	0.888
Heart rate		69.8 ± 12.0	69.1 ± 10.1	0.451
Clinical presentation				
	Unstable angina	232 (92.1)	227 (90.1)	0.435
	NSTEMI	13 (5.2)	12 (4.8)	0.837
	STEMI	7 (2.8)	13 (5.2)	0.171
Medication during hospitalization				
	Oral anticoagulants	3 (1.2)	5 (2.0)	0.476
	Aspirin	249 (98.8)	252 (100)	0.082
	${\mathrm{P2Y}}_{12}$ inhibitor	248 (98.4)	248 (98.4)	1.000
	β-blocker	216 (85.7)	225 (89.3)	0.225
	CCB	79 (31.3)	70 (27.8)	0.380
	Nitrate	192 (76.2)	199 (79.0)	0.455
	Nicorandil	83 (32.9)	70 (27.8)	0.208
	ACEI/ARB	107 (42.5)	106 (42.1)	0.928
	Statins	249 (98.8)	246 (97.6)	0.313
**Angiographic and Procedural characteristics**				
Lesion characteristics				
Left main		13 (5.2)	12 (4.8)	0.837
Bifurcation		51 (20.3)	65 (25.8)	0.138
Chronic total occlusion		118 (46.8)	106 (42.1)	0.282
Severe calcification		33 (13.1)	29 (11.5)	0.588
Severe tortuosity		37 (14.7)	40 (15.9)	0.710
Anticoagulation drug				0.737
Heparin		247 (98.0)	248 (98.4)	
Bivalirudin		5 (2.0)	4 (1.6)	
Heparin dose (mg)		80.3 ± 24.1	81.1 ± 24.9	0.707
Procedure duration (min)		72.4 ± 50.0	69.4 ± 42.5	0.451
Contrast volume (mL)		211.0 ± 86.1	210.6 ± 91.7	0.962
Radiation dose (mGy)		2774.9 ± 2266.2	2447.6 ± 1485.2	0.057
Systolic pressure in sheath		148.2 ± 22.1	143.7 ± 24.1	0.032
Diastolic pressure in sheath		78.1 ± 11.0	76.6 ± 11.5	0.149
Ultrasound assessment time (h)		15.2 ± 4.0	15.2 ± 3.9	0.893
Postoperative LMWH		95 (37.7)	81 (32.1)	0.191
S/A		1.22 ± 0.22	1.12 ± 0.20	<0.001
S/A >1		224 (88.9)	173 (68.7)	<0.001

Values are mean ± SD or n (%). ACEI/ARB, angiotensin converting enzyme inhibitor/angiotensin-receptor blocker; BMI, body mass index; CCB, calcium channel blocker; CABG, coronary artery bypass grafting; NSTEMI, non-ST-elevation myocardial infarction; STEMI, ST-elevation myocardial infarction; TRI, transradial coronary intervention; LMWH, low-molecular-weight heparin; S/A, ratio of the sheath outer diameter to the radial artery inner diameter.

### 3.3 Clinical Outcomes

**Fig. 2.**
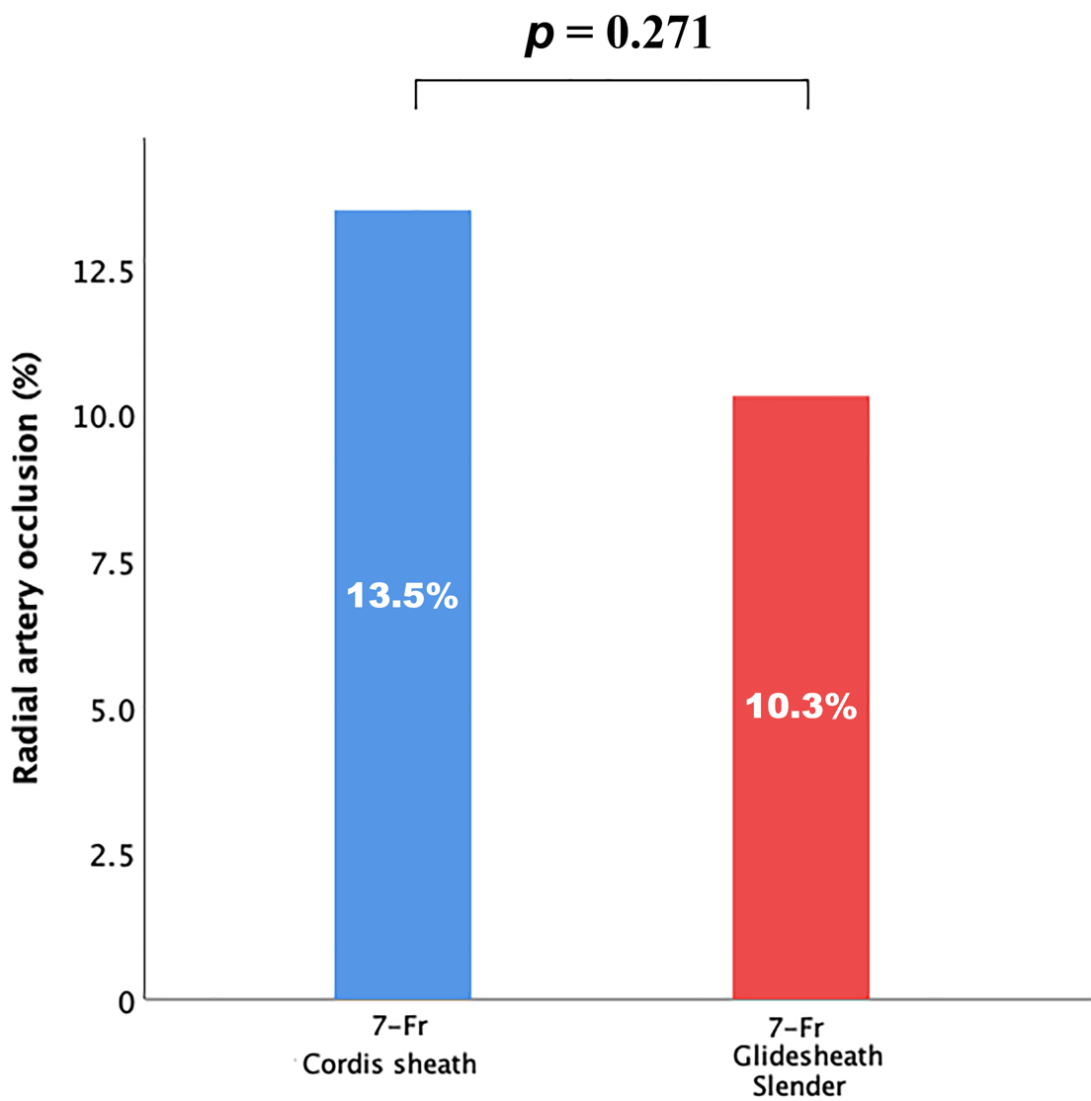
**Incidence of peri-procedural radial artery occlusion by 7-Fr Glidesheath Slender or 7-Fr Cordis sheath**.

By ITT analysis, the primary endpoint of early RAO, as assessed by Ultrasound Doppler imaging in 24 hours after procedure, occurred in 26 (10.3%) patients in the 7-Fr Glidesheath Slender group vs. 34 (13.5%) patients in the 7-Fr Cordis sheath group (*p* = 0.271) (Table 2 and Fig. 2). There were no symptoms in any of the patients, and there was no evidence of acute hand ischemia. Although statistical superiority was not achieved, 7-Fr Glidesheath Slender was associated to a numerically low incidence of peri-procedural RAO compared to 7-Fr Cordis conventional sheath.

**Table 2.** **Clinical outcome and ultrasound-doppler parameters parameters related to the randomized 7-Fr sheath**.

Variables		7-Fr Cordis conventional sheath (n = 252)	7-Fr Glidesheath Slender (n = 252)	*p* value
Clinical outcome				
	Radial artery occlusion	34 (13.5)	26 (10.3)	0.271
	Procedural success*	236 (93.7)	234 (92.9)	0.722
	Pain during the procedure†	2.45 ± 0.95	2.27 ± 0.75	0.017
	Local hematoma	8 (3.2)	7 (2.8)	0.622
	Radial spasm	12 (4.8)	6 (2.4)	0.150
	Arteriovenous fistula	0 (0)	1 (0.4)	1.000
	Pseudoaneurysm	0 (0)	0 (0)	-
	Compartment syndrome	0 (0)	0 (0)	-
Ultrasound-Doppler parameters				
Pre-procedural				
	Diameter of right radial artery, mm	2.55 ± 0.44	2.57 ± 0.47	0.659
	Maximum velocity, cm/s	78.96 ± 24.01	80.06 ± 25.28	0.617
	Minimum velocity, cm/s	16.12 ± 6.18	16.58 ± 7.23	0.449
	Average velocity, cm/s	10.24 ± 6.80	10.73 ± 7.79	0.448
	Resistance index	0.79 ± 0.06	0.79 ± 0.06	0.941
	Volume of blood flow, mL/s	0.03 ± 0.02	0.03 ± 0.02	0.503
Post-procedural				
	Diameter of right radial artery, mm	3.05 ± 0.43	2.97 ± 0.44	0.036
	Maximum velocity, cm/s	70.90 ± 24.57	78.56 ± 28.1	0.003
	Minimum velocity, cm/s	14.50 ± 6.32	16.29 ± 8.20	0.010
	Average velocity, cm/s	8.07 ± 6.20	9.50 ± 7.33	0.028
	Resistance index	0.79 ± 0.06	0.79 ± 0.07	0.651
	Volume of blood flow, mL/s	0.03 ± 0.03	0.04 ± 0.03	0.249

Values are mean ± SD or n (%). *Defined as achievement of final diameter stenosis of <50% by visual estimation and postprocedural TIMI (Thrombolysis In Myocardial Infarction) flow grade 3 in all treated lesions. †Defined as maximal perceived pain during sheath insertion by patients statement according to a numeric rating scale (NRS) going from 0 to 10.

**Fig. 3.**
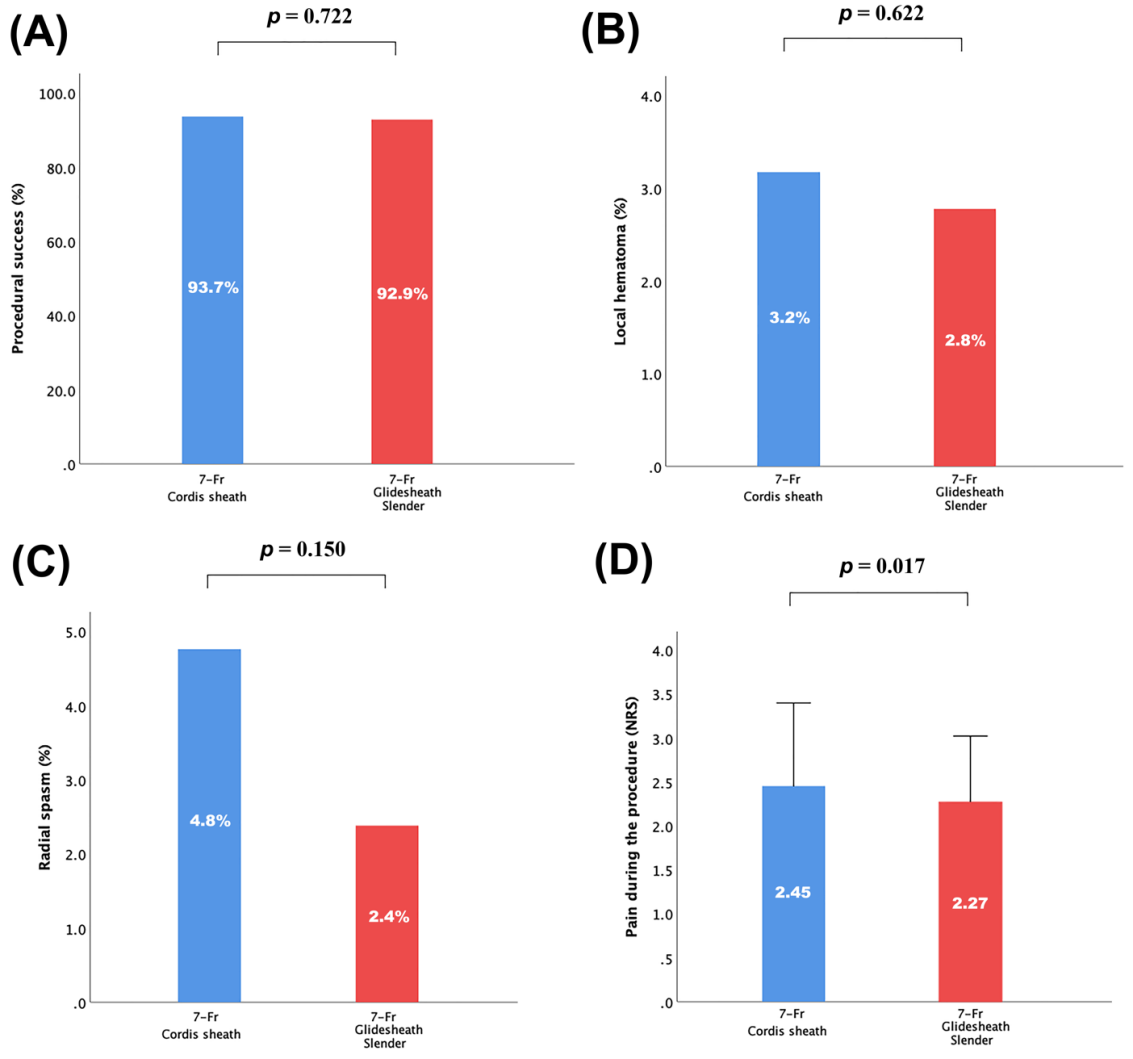
**Incidence of other peri-procedural clinical outcome parameters by 7-Fr Glidesheath Slender or 7-Fr Cordis sheath**. (A) Procedural success. (B) Local hematoma. (C) Radial spasm. (D) Pain during the procedure (NRS).

Procedural success was achieved in 93% of patients. No difference was observed between 7-Fr Glidesheath Slender and 7-Fr Cordis sheath regarding procedural success (93.7% vs. 92.9%; *p* = 0.722) (Fig. 3A). Other secondary endpoints of access-site complications, including local hematoma, radial spasm, and arteriovenous fistula were not statistically different between the two groups (Fig. 3B,C). Although non-significant, the incidence of local hematoma and radial spasm in 7-Fr Glidesheath Slender group were both numerically lower than that of the 7-Fr Cordis sheath group. Besides, use of the Glidesheath Slender was associated with significantly less pain during the procedure (NRS, 2.27 ± 0.75 vs. 2.45 ± 0.95, *p* = 0.017) (Fig. 3D). In both groups, there were no incidences of pseudoaneurysm and compartment syndrome. Similar results of clinical outcome parameters were found in the ATS population (**Supplementary Table 1**).

Table 2 and **Supplementary Fig. 4** shows the radial artery ultrasound examination parameters before and after TRI. Despite the radial artery diameter in both groups were larger after procedure, the postoperative radial artery diameter of 7-Fr Glidesheath Slender group was significantly smaller than the 7-Fr Cordis sheath group (2.97 ± 0.44 vs. 3.05 ± 0.43 mm; *p* = 0.036). In addition, all parameters of blood flow velocity (e.g., maximum velocity [78.6 ± 28.1 vs. 70.9 ± 24.6 cm/s], minimum velocity [16.3 ± 8.2 vs. 14.5 ± 6.3 cm/s], and average velocity [9.5 ± 7.3 vs. 8.1 ± 6.2 cm/s]) in Glidesheath Slender group were significantly higher compared with Cordis sheath group after TRI (all *p *< 0.05). However, no significant difference was found between the two groups in terms of resistance index or volume of blood flow. An exploratory comparison of ultrasound-Doppler parameters of radial artery related to the randomized 7-Fr sheath was performed and showed similar results between the 2 groups by ATS analysis) (**Supplementary Table 2**).

### 3.4 Independent Predictors of Early RAO

BMI, peripheral artery disease, heparin dose, the ratio of S/A, preoperative radial artery diameter, CCB use, and volume of blood flow were significantly associated with RAO in univariable logistic regression analysis (**Supplementary Table 3**). In multivariable regression analysis, independent predictors of RAO were body mass index, peripheral artery disease, heparin anticoagulation, calcium channel blockers use, statins use, and ratio of S/A (**Supplementary Table 4**).

## 4. Discussion

**Fig. 4.**
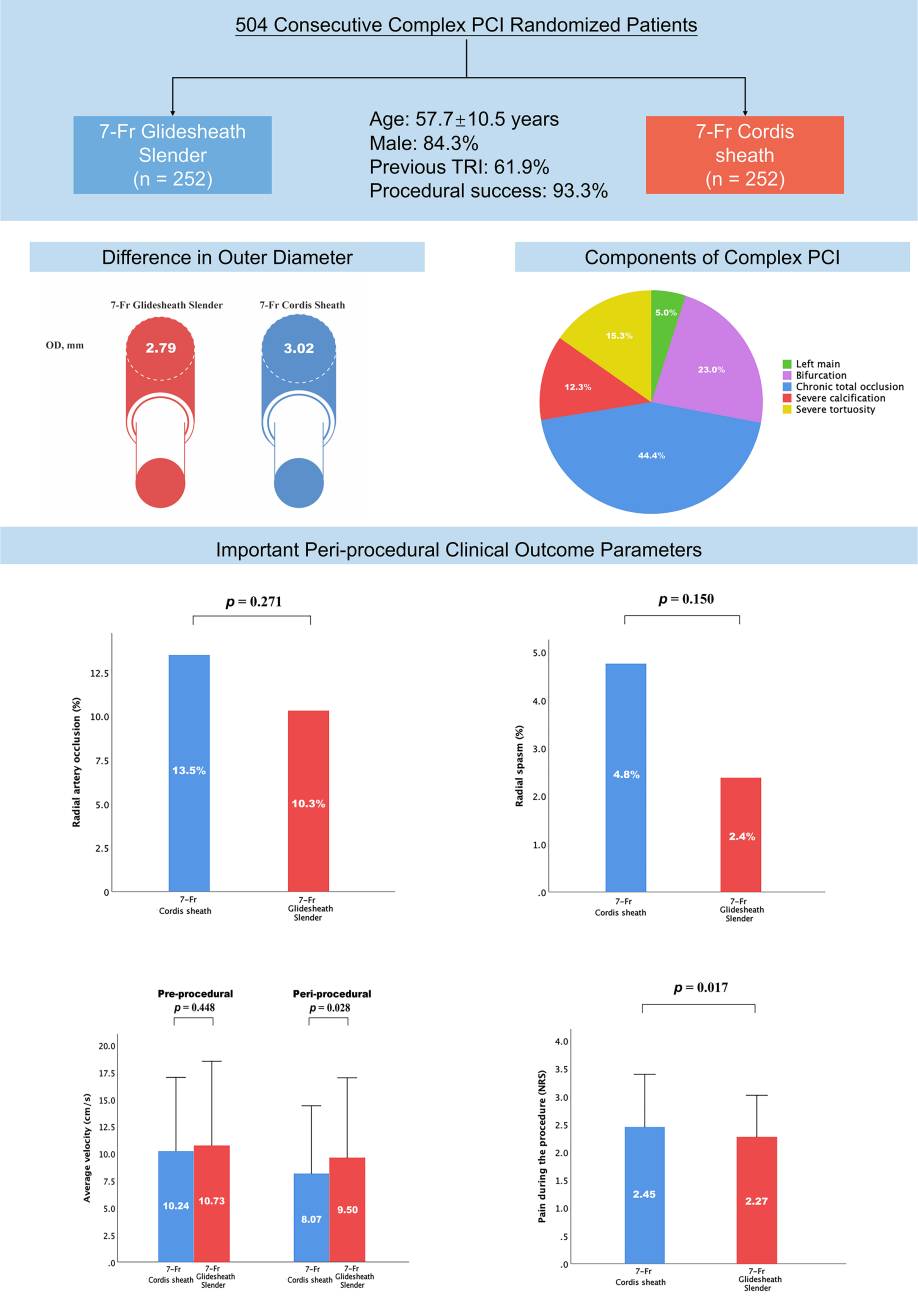
**Patient Characteristics, Study Groups, and Important Peri-procedural Clinical Outcome Parameters After PCI of Complex Coronary Lesions Related to the Randomized 7-Fr Sheaths**.

Our study represents the first randomized trial comparing the efficacy of 7-Fr TRA access with Glidesheath Slender versus Cordis sheath for PCI of complex coronary lesions (Fig. 4). The main findings of the present study can be summarized as follows: (1) the trial failed to demonstrate superiority of 7-Fr Glidesheath Slender for the prevention of early RAO evaluated using Doppler ultrasound within 24 hours after complex PCI procedures through TRA compared with 7-Fr Cordis sheath. (2) Although the 7-Fr Glidesheath Slender was associated with reduced risk of early RAO, the trial did not have enough power to detect a meaningful difference in efficacy outcomes; (3) 7-Fr Glidesheath Slender reduced the pain during the procedure and increase the blood flow velocity of the radial artery assessed by ultrasound, although rates of local hematoma and radial spasm were similar between the groups; (4) routine 7-Fr Glidesheath Slender may not be considered mandatory in RAO prevention in large-bore complex transradial PCI and need to be confirmed in a large-scale randomized trial.

Treatment of complex coronary lesions often required specific equipment, such as true bifurcation lesions treated with double-stent technique, left main lesions, heavy calcified lesions requiring rotational atherectomy, severe tortuosity, and CTO lesions requiring several devices simultaneously in one guiding catheter. The use of large-bore sheaths has been documented in 60% to 70% of CTO TRI instances [17, 18]. What’s more, femoral artery cannot be used as operative approach in some patients with severe peripheral vascular disease at lower limbs, neither in some patients with cardiac dysfunction who need to use femoral artery for hemodynamic support, making transradial 7-Fr PCI a rigid demand.

It is known that the use of a large-bore (≥7-Fr) guiding catheter throught TRA approach is limited by the small size of radial artery diameter; therefore, its use has been associated with increased risks of RAO because of sheath to-artery mismatch and subsequent vascular injury [19, 20]. In another study, we performed peri-procedural radial artery ultrasound in a cohort of 130 patients who had undergone TRA catheterization via a standard 7-Fr sheath and found asymptomatic early RAO in 11 cases (8.5%) [14]. Notably, 7-Fr Glidesheath Slender, for example, is a newly created 7-Fr compatible thin-walled sheath with the outer diameter of a 6-F sheath, reflecting a 1-F reduction of the OD over standard 7-Fr sheaths [13]. In this regard, it is necessary to conduct randomized comparison between the 7-Fr Glidesheath Slender and Cordis sheath in patients undergoing complex PCI through TRA approach.

In our study, it was revealed that 7-Fr Glidesheath slender was associated to a relatively low peri-procedural RAO (within 12–24 hours) following 7-French TRI compared to standard 7-Fr radial sheaths (10.3% vs. 13.5%, *p* = 0.271). Previously, a multicenter trial involving 60 consectutive patients have evaluated the safety and feasibility of the 7-Fr Glidesheath slender for complex transradial PCI, which showed a rate of 1-month RAO of 4.8% despite of high procedural success [13]. A prospective multicentre study recruiting patients undergoing left distal transradial approach for coronary CTO interventions using a 7-Fr Glidesheath Slender found the rate of left distal RAO of 4.3% at the 1-month decteced by Doppler ultrasound [21]. Moreover, previous studies have shown that Braidin slender 7-Fr sheath had a RAO rate of 5.8% at 1 month of follow-up in 154 patients with left main bifurcation diseases [22]. Isawa *et al*. [23] reported that in the matched patients, the use of 7-Fr Glidesheath slender was significantly less likely to develop ultrasound diagnosed RAO (1.4% vs. 4.1%) at 30 days compared to the use of 7.5-Fr Sheathless system. Notably, the incidence of RAO ranged from a 7.5% incidence during the first 24 hours to 5.5% at 30 days of follow-up [15, 24]. RAO is currently recognized as a barrier to re-intervention in the same radial artery and limits its use as an arterial conduit in patients undergoing coronary artery bypass grafting, or creating an arteriovenous fistula in patients requiring hemodialysis; therefore, prevention of RAO is clinically significant when TRA approach is used.

All participants in our study assessed RAO by Doppler ultrasound within 24 hours. Early dection of RAO at this stage has been considered as the gold-standard method to identify both thrombotic obstruction and lack of flow, where strategies (such as patent hemostasis, adequate anticoagulation and postprocedural hemostatic care) to reduce the incidence of RAO could be implemented [25, 26]. In addition, confirmation of radial artery patency before hospital discharge are suggested to be part of post-PCI patients after TRA approach. As a result, in patients who have invasive procedures through the radial artery, we advocate undertaking an early RAO examination (preferably within 24 hours after PCI or before discharge).

It was reported that 7-Fr Glidesheath slender has the potential to minimize radial artery complications in complex PCI [13]. The use of the hydrophilic coated Slender sheath during radial coronary angiography or PCI was related with decreased pain during sheath insertion [27]. Thus, it seemed reasonable that smaller sheath and thinner sheath coat not only reduce the incidence of early RAO, but also alleviate pain and spasm. The main findings in our study supports this hypothesis. The pain during the procedure in the 7-Fr Glidesheath slender group in our study is significantly lower than that of the Cordis group. Additionally, postoperative ultrasound-doppler assessment of radial artery showed that, patients in the Glidesheath slender group had an apparently higher blood flow velocity than those the standard 7-Fr radial sheath group. This difference may be attributed to the smaller OD of the 7-Fr Glidesheath slender, thus avoid the injury to the radial artery and reduce the inflammation-mediated thickening of the intimal-medial layer.

With regard to the risk factor of RAO, previous trials have demonstrated that RAO may be related to the following factors such as older age, female gender, longer procedure times, previous TRI, insufficient unfractionated heparin, narrow radial artery, and S/A >1 [15]. In a Japanese study using Ultrasound Doppler assessment of the radial artery, the S/A ratio >1 was associated with lower blood flow in radial artery after TRI, but the association with RAO has not been confirmed [28]. In addition, Fan *et al*. [16] found that the 7-Fr sheaths were related with a greater incidence of RAO in 248 participants randomized to 6- Fr or 7-Fr sheaths (5.71% vs. 7.34%, *p *< 0.01). In our study, multivariate regression analysis showed that several baseline characteristics associated with an increased risk of RAO, such as lower BMI, peripheral artery disease and higher S/A value. In contrast, several factors were shown to reduce the rate of RAO, including the use of heparin, CCB and statins. Other characteristics previously reported to influence the incidence of RAO, including as age, previous TRI, hemostatic compression, and procedure time, were not independent predictors of early RAO after 7-Fr TRI in this study.

Prevention of RAO is important from a clinical standpoint and should be a top priority [29]. Recently, an International Consensus Paper summarized RAO prevention strategies following percutaneous TRA diagnostic or interventional procedures [30], which included (1) reducing the size of the sheath or catheter; (2) applying adequate procedural anticoagulation; (3) achieving nonocclusive hemostasis; (4) using a minimal pressure strategy together with shortened hemostasis time (≤2 hours); (5) Pre-puncture subcutaneous nitrates and post-procedural pre-hemostasis intra-arterial nitrates; (6) systematic assessment of radial artery patency with ultrasound before hospital discharge. Therefore, increasing the adoption of simple and effective methods to reduce RAO incidence are essential to increase the likelihood of access in case of repeat catheterization or coronary artery bypass grafting surgery.

This study has several limitations. First, The RAO rate in the 7-Fr slender group (10.3%) in our study was relatively lower than standard 7-Fr group (13.5%). Our original sample size calculation was based on the first prospective registry of complex TRI cases using the 7-Fr Glidesheath Slender to determine the rate of RAO [14], in which RAO (4.8%) was assessed at 1 month. Resource limitations of the rate of periprocedural RAO after complex PCI prevented us from conducting an event-driven sample size consideration in more accurate pattern. Second, in our study, RAO was assessed during the first 24 hours after complex PCI with TRA access and long-term radial artery patency was not evaluated. This may partly explain the higher rate for periprocedural RAO in the Glidesheath Slender group. However, early detection of RAO in the first 24 hours after TRI is conducive to reopen the radial artery timely [31]. Fourth, local hematomas was not graded in a specific scale (range from type I to type IV) according to the EASY criteria [32]. Fifth, although the average of radial puncture attempt ranged from 1 to 3 times by high-volume and experienced operators, the specific number of radial puncture attempts was not documented in this study. Sixth, this is a single-center study in an academic referral institution with complex PCI expertise [33, 34], and our results may not be generalized to all patients undergoing PCI in other centers and broader populations. Hence, these results will need to be corroborated in multi-centers with more cases in 7-Fr TRI. Finally, other TRA best practices, such as patent hemostasis, minimizing compression time and ipsilateral ulnar compression were not systematically implemented. The higher rate for periprocedural RAO in the Glidesheath Slender group could reflect the superiority criteria for the primary endpoint were not met in the overall trial.

## 5. Conclusions

In this prospective, randomized, comparative trial designed to explore the superiority in periprocedural RAO prevention using 7-Fr Glidesheath Slender through TRA for patients undergoing PCI of complex coronary lesions, 7-Fr Glidesheath Slender was not superior to conventional 7-Fr in the prevention of periprocedural RAO within 24 hours following complex PCI, without reducing RAO occurrence. Evaluation of early RAO after complex TRI could facilitate physicians to recanalize RAO by pharmacological and invasive approach for potential future cardiac catheterizations. In aggregate, routine 7-Fr Glidesheath Slender may not be considered mandatory in RAO prevention in large-bore complex transradial PCI and need to be confirmed in a large-scale randomized trial.

## Supplementary Material

Supplementary material associated with this article can be found, in the online version, at https://doi.org/10.31083/j.rcm2310329.
