# Supplementary material for: Effect of Thin-Walled Radial Sheath for Large-Bore Access On Reducing Periprocedural Radial Artery Occlusion Following Complex PCI: The REDUCE-RAO Randomized Trial
Source: Rev Cardiovasc Med. 2022 Sep 28;23(10):329. doi: 10.31083/j.rcm2310329 (PMC11267382; doi:10.31083/j.rcm2310329)
Supplement: Supplementary file 1 [file 2153-8174-23-10-329-s1.docx]

**Supplementary Materials**

Supplementary Table 1. Clinical outcome parameters related to the randomized 7-Fr sheath in as-treated set population (N=500).

| Variables | 7-Fr Cordis conventional sheath (n=251) | 7-Fr Glidesheath Slender (n=249) | P value |
| --- | --- | --- | --- |
| Radial artery occlusion | 34 (13.5) | 25 (10.0) | 0.224 |
| Procedural success* | 235 (93.6) | 231 (92.8) | 0.704 |
| Pain during the procedure (NRS) | 2.45 ± 0.95 | 2.29 ± 0.73 | 0.029 |
| Local hematoma | 8 (6.9) | 7 (5.5) | 0.643 |
| Radial spasm | 11 (4.4) | 5 (2.0) | 0.131 |
| Arteriovenous fistula | 0 (0) | 1 (0.4) | 0.315 |
| Pseudoaneurysm | 0 (0) | 0 (0) | 1.000 |
| Compartment syndrome | 0 (0) | 0 (0) | 1.000 |

Values are mean ± SD or n (%). *Deﬁned as achievement of ﬁnal diameter stenosis of <50% by visual estimation and postprocedural TIMI (Thrombolysis In Myocardial Infarction) ﬂow grade 3 in all treated lesions. †Defined as maximal perceived pain during sheath insertion by patients statement according to a numeric rating scale (NRS) going from 0 to 10.

Supplementary Table 2. Ultrasound-Doppler parameters of radial artery related to the randomized 7-Fr sheath in as-treated set population (N=500).

| Variables | 7-Fr Cordis conventional sheath (n=251) | 7-Fr Glidesheath Slender (n=249) | P value |
| --- | --- | --- | --- |
| Pre-procedural |  |  |  |
| Radial artery diameter (mm) | 2.56 ± 0.44 | 2.58 ± 0.47 | 0.577 |
| Maximum velocity (cm/s) | 79.0 ± 24.1 | 80.3 ± 25.3 | 0.542 |
| Minimum velocity (cm/s) | 16.1 ± 6.2 | 16.6 ± 7.3 | 0.430 |
| Average velocity (cm/s) | 10.2 ± 6.8 | 10.8 ± 7.8 | 0.408 |
| Resistance index | 0.79 ± 0.06 | 0.79 ± 0.06 | 0.987 |
| Volume of blood flow (ml/s) | 0.03 ± 0.02 | 0.03 ± 0.02 | 0.475 |
| Peri-procedural |  |  |  |
| Radial artery diameter (mm) | 3.05 ± 0.42 | 2.97 ± 0.44 | 0.029 |
| Maximum velocity (cm/s) | 71.0 ± 24.6 | 78.6 ± 28.5 | 0.003 |
| Minimum velocity (cm/s) | 14.5 ± 6.3 | 16.3 ± 8.2 | 0.011 |
| Average velocity (cm/s) | 8.1 ± 6.2 | 9.5 ± 7.3 | 0.030 |
| Resistance index | 0.79 ± 0.06 | 0.79 ± 0.07 | 0.687 |
| Volume of blood flow (ml/s) | 0.03 ± 0.03 | 0.04 ± 0.03 | 0.278 |

Values are mean ± SD or n (%).

Supplementary Table 3. Univariable predictors of RAO (N=504).

|  | Odds ratio (95% CI) | *P* value |
| --- | --- | --- |
| Age | 1.016 (0.990-1.043) | 0.239 |
| Male | 0.711 (0.359-1.409) | 0.328 |
| BMI, per 1 unit decrease | 1.158 (1.048-1.279) | 0.004 |
| Unstable angina | 3.110 (0.734-13.181) | 0.124 |
| Current smoker | 0.681 (0.397-1.168) | 0.163 |
| Hypertension | 0.896 (0.516-1.554) | 0.695 |
| Diabetes mellitus | 1.361 (0.783-2.366) | 0.275 |
| Previous myocardial infarction | 1.053 (0.572-1.938) | 0.869 |
| Previous PCI | 1.494 (0.868-2.570) | 0.147 |
| Previous CABG | 3.746 (0.334-41.197) | 0.284 |
| Previous TRI | 1.646 (0.910-2.977) | 0.100 |
| Peripheral artery disease | 2.692 (1.150-6.302) | 0.022 |
| Left main disease | 1.927 (0.695-5.342) | 0.207 |
| Bifurcation lesions | 0.817 (0.418-1.597) | 0.555 |
| Chronic total occlusion | 1.500 (0.873-2.576) | 0.142 |
| Severe calcification | 0.475 (0.166-1.360) | 0.166 |
| Severe tortuosity | 0.706 (0.308-1.616) | 0.410 |
| Heparin anticoagulation | 0.260 (0.063-1.069) | 0.062 |
| Heparin dose | 0.984 (0.973-0.996) | 0.011 |
| Procedure duration | 1.004 (0.999-1.010) | 0.109 |
| Contrast volume (ml) | 1.002 (0.999-1.005) | 0.184 |
| Systolic pressure in sheath | 0.992 (0.980-1.004) | 0.170 |
| Diastolic pressure in sheath | 0.984 (0.960-1.008) | 0.197 |
| Successful PCI | 0.490 (0.204-1.181) | 0.112 |
| Compression time | 1.017 (0.950-1.090) | 0.624 |
| Postoperative LMWH | 0.847 (0.475-1.510) | 0.574 |
| S/A, per 0.1 increase | 1.276 (1.131-1.440) | <0.001 |
| S/A>1 | 2.201 (0.970-4.993) | 0.059 |
| Oral anticoagulants | 3.842 (0.935-15.786) | 0.062 |
| β-blocker use | 2.145 (0.750-6.135) | 0.154 |
| CCB use | 0.498 (0.251-0.987) | 0.048 |
| Statins use | 0.260 (0.063-1.069) | 0.062 |
| Nitrate | 1.051 (0.547-2.020) | 0.881 |
| Nicorandil | 0.815 (0.444-1.495) | 0.508 |
| ACEI/ARB | 0.973 (0.563-1.680) | 0.921 |
| Preoperative radial artery diameter | 0.302 (0.153-0.597) | 0.001 |
| Maximum velocity | 1.001 (0.991-1.012) | 0.788 |
| Minimum velocity | 0.957 (0.911-1.005) | 0.079 |
| Average velocity | 0.960 (0.919-1.003) | 0.069 |
| Volume of blood flow, per 0.1 increase | 0.160 (0.034-0.746) | 0.020 |
| Pain during the procedure (NRS) | 1.009 (0.738-1.379) | 0.957 |
| Local hematoma | 1.895 (0.519-6.917) | 0.333 |
| Radial spasm | 0.922 (0.207-4.115) | 0.916 |
| 7-Fr Glidesheath Slender | 0.738 (0.428-1.270) | 0.272 |

ACEI/ARB, angiotensin converting enzyme inhibitor/angiotensin-receptor blocker; BMI: body mass index; CCB, calcium channel blocker; CABG, coronary artery bypass grafting; LMWH, low-molecular-weight heparin; NSTEMI, non-ST-elevation myocardial infarction; STEMI, ST-elevation myocardial infarction; S/A, ratio of the sheath outer diameter to the radial artery inner diameter; RAO, radial artery occlusion; TRI, transradial coronary intervention.

Supplementary Table 4. Multivariable predictors of RAO.

|  | Odds ratio (95% CI) | *P* value |
| --- | --- | --- |
| BMI, per 1 unit decrease | 1.149 (1.033-1.728) | 0.011 |
| Peripheral artery disease | 2.860 (1.140-7.177) | 0.025 |
| Heparin anticoagulation | 0.187 (0.041-0.857) | 0.031 |
| CCB use | 0.466 (0.227-0.958) | 0.038 |
| Statins use | 0.178 (0.040-0.801) | 0.024 |
| S/A, per 0.1 increase | 1.256 (1.106-1.426) | <0.001 |

Multivariable Logistic regression analysis was performed using a backward stepwise method, including the baseline variables with P<0.10 in the univariable analysis and any other baseline variables judged to be of clinical relevance from previously published literature, speciﬁcally: BMI, diabetes, previous TRI, peripheral artery disease, heparin anticoagulation, procedure duration, successful PCI, S/A, CCB use, statins use, compression time, preoperative radial artery diameter, volume of blood flow, and 7-Fr Glidesheath Slender.

RAO, radial artery occlusion; other abbreviations as in Table 1 and 2.


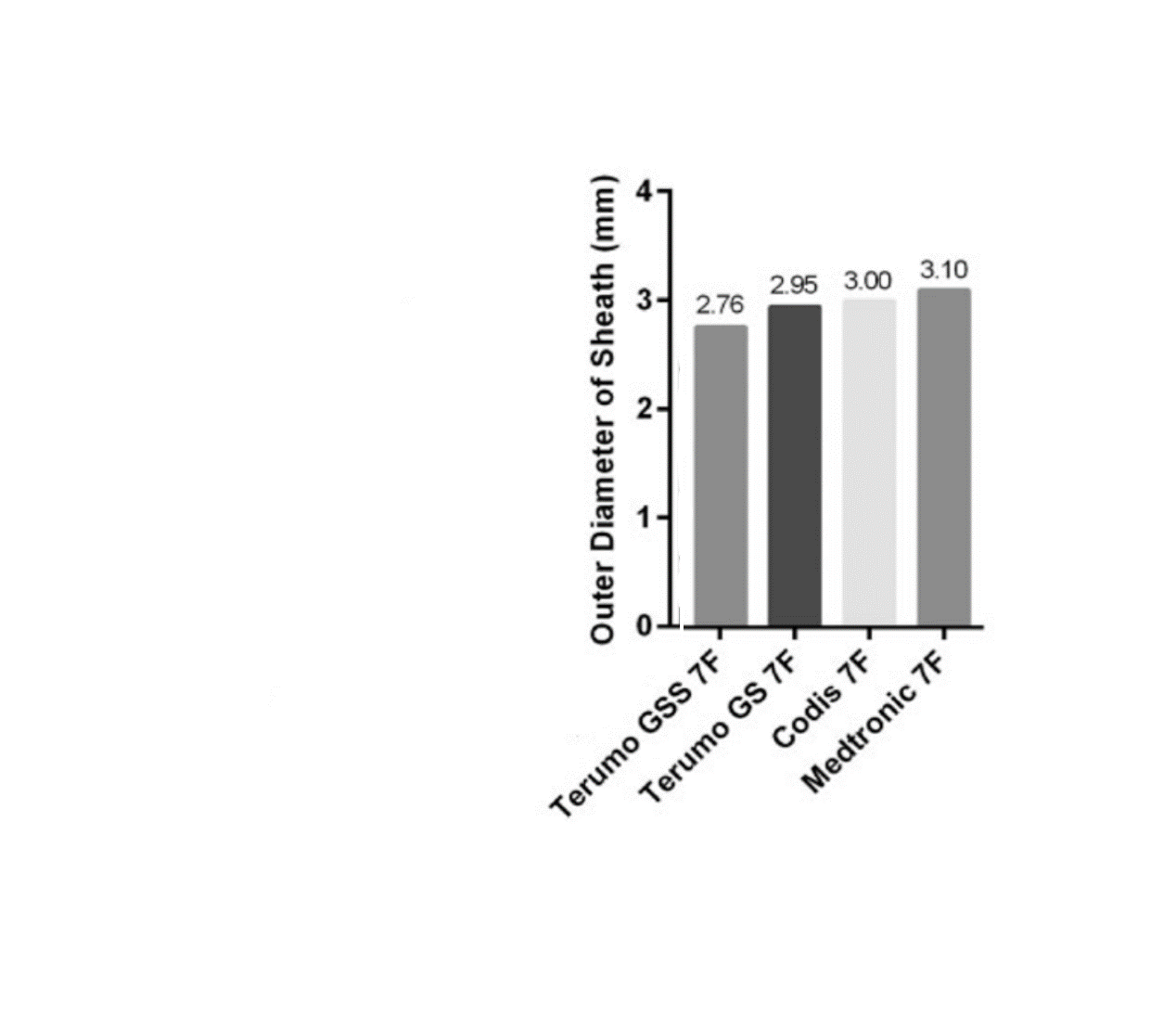


Supplementary Fig. 1. Comparison of 7 Fr sheaths outer diameter by manufacturer.

**
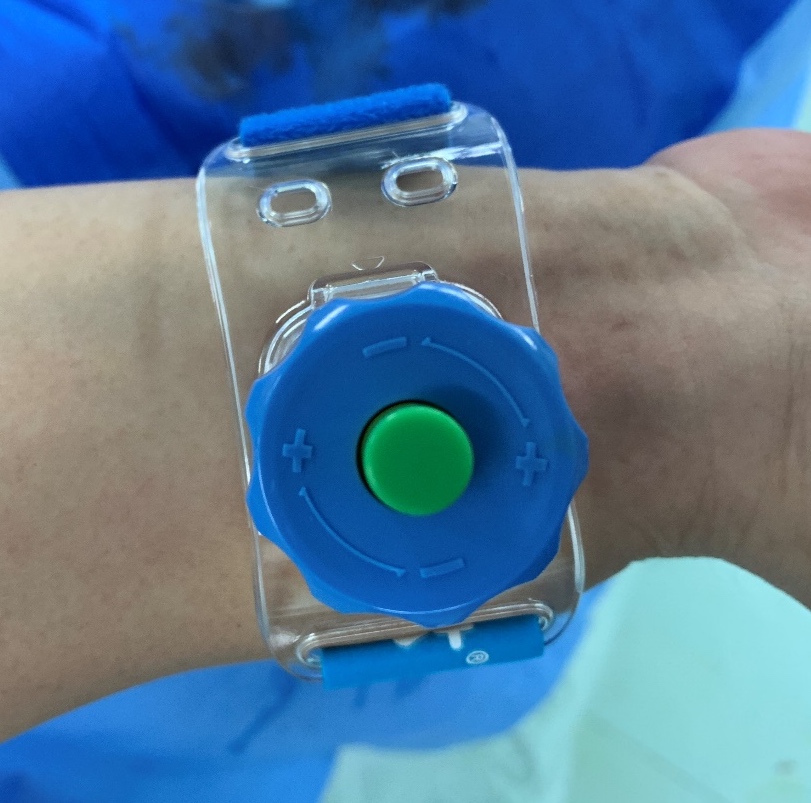
**

Supplementary Fig. 2. The radial artery puncture medical helix compression device that was used in this trail.

| 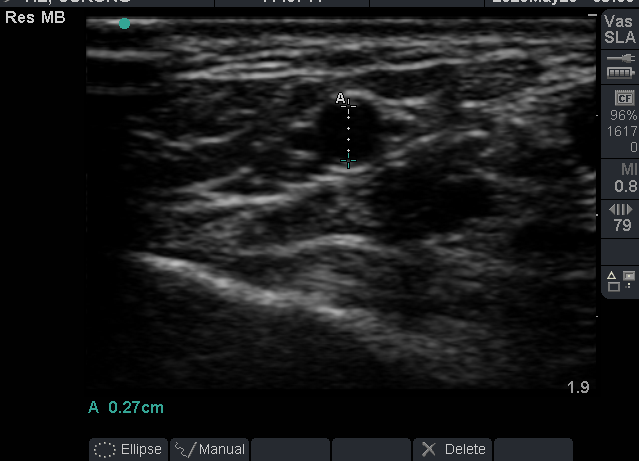 | 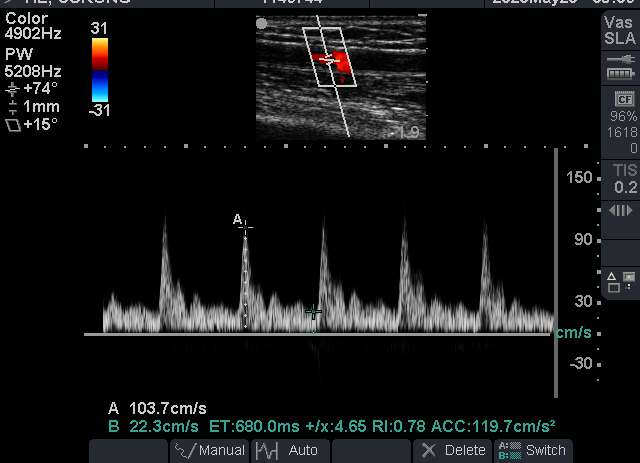 |
| --- | --- |

Supplementary Fig. 3. Ultrasound-Doppler assessment of radial artery.


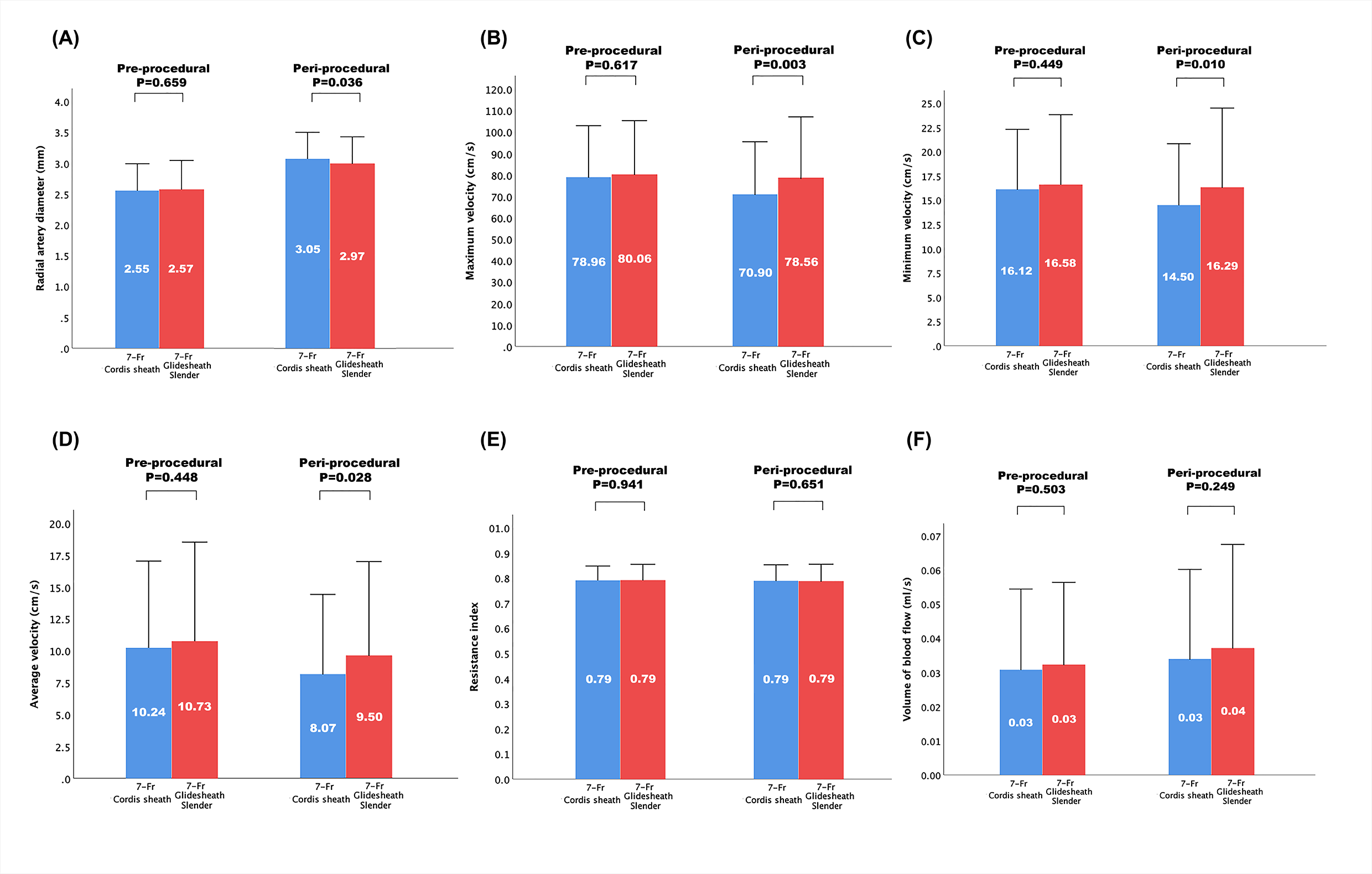


Supplementary Fig. 4. Ultrasound-Doppler parameters of radial artery by 7-Fr Glidesheath Slender or 7-Fr Cordis sheath. (A) Radial artery diameter. (B) Maximum velocity. (C) Minimum velocity. (D) Average velocity. (E) Resistance index. (F) Volume of blood flow.
